# Implementation of an innovative virtual selective screening program for early detection of cerebral palsy in British Columbia

**DOI:** 10.3389/fpubh.2026.1754120

**Published:** 2026-03-02

**Authors:** Keith O'Connor, Nandy Fajardo, Carol Lai, Vivian Wong, Mor Cohen-Eilig, Ram A. Mishaal

**Affiliations:** 1 Department of Occupational Therapy, British Columbia Children's Hospital, Vancouver, BC, Canada; 2 Occupational Science and Occupational Therapy, The University of British Columbia, Vancouver, BC, Canada; 3 British Columbia Children's Hospital, Vancouver, BC, Canada; 4 The University of British Columbia, Vancouver, BC, Canada; 5 Rehabilitation Science Graduate Program, The University of British Columbia, Vancouver, BC, Canada; 6 Developmental Medicine, British Columbia Children's Hospital, Vancouver, BC, Canada; 7 Department of Pediatrics, Faculty of Medicine, University of British Columbia, Vancouver, BC, Canada

**Keywords:** cerebral palsy, early detection, general movement assessment (GMA), health system innovation, high-risk infant, telehealth, virtual screening

## Abstract

Early identification of cerebral palsy (CP) enables timely intervention during critical periods of neuroplasticity, yet diagnosis remains delayed in many health systems. In British Columbia (BC), fragmented referral pathways and restrictive neonatal follow-up criteria have limited access to standardized early motor assessment, particularly for infants outside major urban centers. To address these gaps, a province-wide virtual screening pathway was developed to improve equitable access to early CP detection. The Early Motor Screening Program (EMSP) was implemented in 2022 as a virtual, risk-stratified screening pathway for infants at elevated neurological risk. The program integrates caregiver-recorded General Movements Assessment (GMA) videos, telehealth-based clinical review, and coordinated referral to an interdisciplinary Cerebral Palsy Early Detection Clinic (CPEDC). Eligibility criteria were defined using epidemiological risk stratification, prioritizing infants with medical conditions associated with substantially increased CP risk. This community-based implementation case study used a descriptive observational design drawing on routinely collected program and clinical data. Outcomes included program uptake, screening completion, GMA findings, referral patterns, caregiver experience, and system-level indicators. Analyses were descriptive and interpreted as implementation and pathway outcomes rather than diagnostic performance. Between June 2022 and September 2025, 883 infants were referred to the EMSP, with 622 (70%) completing GMA assessments. Abnormal GMA findings were identified in 114 infants (18%), who were referred to CPEDC for further evaluation. Among these infants, 25 (22%) received a CP diagnosis, while others required monitoring or were discharged. Referral volumes increased steadily across the study period, with participation from all health authorities and substantial engagement from rural and remote regions. Caregiver feedback indicated high acceptability and feasibility of the virtual screening model. Provincial data demonstrated a temporal, system-level reduction in the average age of CP diagnosis among high-risk infants engaged in early detection pathways, from approximately 25 months to 7 months. This case study demonstrates the feasibility of implementing a province-wide virtual early motor screening pathway within a publicly funded health system. Coordinated, risk-based, and telehealth-enabled models may expand access to early CP identification and support more timely clinical pathways, with potential applicability to other jurisdictions seeking equitable early neurodevelopmental screening.

## Context: provincial landscape and need for innovation

Cerebral palsy (CP) is the most common cause of motor impairment in childhood (1). It results from disturbances to the developing brain occurring from pregnancy through early infancy and is associated with a broad spectrum of neuromotor impairments, functional severity, concomitant health conditions, and secondary complications (2).

Early diagnosis of CP offers a critical opportunity to optimize developmental outcomes. Timely identification supports cognitive and motor development during a key period of neuroplasticity and enables early access to CP-specific interventions that are most effective in infancy (3, 4). A growing body of evidence demonstrates that interventions provided early in life are associated with greater gains in functional outcomes (4, 5). Early diagnosis also supports family wellbeing and facilitates prevention of secondary complications (6). Despite clinical guidelines recommending diagnosis as early as 3–4 months of age (1), the average age of CP diagnosis in Canada in 2021 remained 18.9 months (2, 8), and was even later in British Columbia (BC), at approximately 25 months.

In BC, no standardized diagnostic pathway for CP existed. As a result, diagnosis was often delayed, with responsibility alternating between medical disciplines such as neurologists and pediatricians until clinical features became sufficiently evident to confirm the diagnosis. These delays limit access to early intervention, contribute to increased parental stress, and may strain the parent–infant relationship (2, 7). Similar patterns have been described in the literature, where diagnosis is frequently delayed due to the absence of hallmark signs or an unremarkable early medical history that might otherwise prompt clinical suspicion (8). Healthcare professionals may further postpone diagnosis due to diagnostic uncertainty and concerns about misclassification (9), and families have reported learning of their child's diagnosis incidentally during unrelated clinical encounters. Collectively, these factors have contributed to a fragmented and reactive approach to early CP identification.

Neonatal follow-up (NFU) programs in BC employ variable and often restrictive eligibility criteria that capture only a subset of infants at elevated risk of CP. Consequently, many high-risk infants did not receive standardized motor screening, including the General Movements Assessment (GMA) (10). This gap mirrors challenges observed internationally, where access to standardized neurodevelopmental assessments and trained providers remains limited (5, 7). In the absence of these tools, clinicians may rely on less sensitive measures or clinical judgement alone, leading to a “wait-and-see” approach and missed opportunities for early detection (11).

The GMA provides a validated and sensitive method for assessing the developing nervous system through observation of spontaneous infant movement (10). Fidgety movements typically emerge between 10- and 20-weeks corrected age (CA), peaking at 12–16 weeks CA. The absence of fidgety movements is one of the strongest early predictors of CP, with reported sensitivity of 97% and specificity of 89% (12, 13).

This Community Case Study outlines the design, implementation, and early outcomes of the Early Motor Screening Program (EMSP), BC's first province-wide virtual screening pathway for infants at elevated neurological risk. By shifting away from traditional clinic-based GMA delivery, the program establishes a coordinated provincial pathway for early CP detection and addresses gaps in access to standardized screening across geographic and service contexts. This study is a community-based implementation case study describing the design, provincial implementation, and early service-level outcomes of the EMSP in BC. The evaluation employed a descriptive observational cohort design drawing on routinely collected clinical and program data generated through real-world service delivery. The primary intent was implementation-focused and hypothesis-generating rather than hypothesis-testing. Specifically, the evaluation aimed to describe (1) uptake and reach of a province-wide virtual GMA screening pathway for high-risk infants, (2) feasibility and timeliness of virtual service delivery, and (3) early clinical and system-level outcomes associated with program implementation.

Analyses were primarily descriptive, including counts, proportions, temporal trends, and summary statistics. No formal diagnostic accuracy testing, causal inference, or economic modeling was undertaken. Observed associations, such as changes in referral volumes or age at diagnosis, should therefore be interpreted as contextual and descriptive rather than causal. Given the pragmatic, real-world nature of the EMSP, findings reflect both clinical practice and health system behavior, including referral patterns, caregiver engagement, and service capacity.

## BC's early detection pathway creation and implementation

Building on the gaps identified in the provincial early detection landscape, British Columbia initiated a multi-year, province-wide collaborative process to strengthen pathways for early identification of cerebral palsy. Between 2017 and 2021, the British Columbia Cerebral Palsy Advisory Committee (BCCPAC) convened clinicians across disciplines (developmental pediatrics, neurology, occupational therapy, physiotherapy), health system leaders, researchers, and caregivers of children with CP to guide the development of an evidence-informed provincial approach to early diagnosis (14). Through structured literature reviews, environmental scans of international diagnostic models, and multidisciplinary consensus informed by clinical expertise and caregiver perspectives, the committee identified the prevailing “watch-and-wait” approach as a major contributor to delayed diagnosis and missed opportunities for early intervention, particularly among infants who did not meet restrictive neonatal follow-up criteria, despite elevated medical risk. In response, the BCCPAC recommended a shift toward a proactive, standardized model of early identification that would ultimately lead to early diagnosis, including expansion of medical risk factor definitions and adaptation of international best-practice frameworks such as the AACPDM Early Detection of CP Care Pathway to the BC context (15).

Comparable pathway-based approaches have been developed internationally. In Europe, the Danish CP-EDIT program integrated neurological examination, General Movements Assessment (GMA), the Hammersmith Infant Neurological Examination, and neuroimaging across both high-risk and broader infant populations to reduce the age of diagnosis irrespective of neonatal risk status (16). In Australia, the Baby Moves smartphone application enabled parents to record infant movement videos for remote GMA, demonstrating the feasibility of scalable, technology-enabled screening beyond specialist clinics (17). European implementation studies have similarly shown that remote GMA can be integrated within clinical follow-up programs to enhance access to early identification (18). Unlike many existing models, which operate within single institutions or research cohorts, the EMSP–CPEDC pathway was designed as a province-wide, publicly funded system of care that integrates risk-based eligibility, virtual screening, and interdisciplinary diagnostic assessment within routine clinical practice.

The Early Motor Screening Program (EMSP) was developed to operationalise these recommendations by translating the BCCPAC's evidence-informed pathway into a province-wide screening model for infants at elevated neurological risk. In designing the program, the AACPDM framework (15) served as the primary reference. A critical gap was identified in the absence of a coordinated provincial approach to screening high-risk infants using the GMA. To ensure that referral pathways were evidence-informed and clinically defensible, the EMSP implemented structured inclusion criteria grounded in epidemiological risk stratification. Medical eligibility was defined using an odds ratio (OR) threshold greater than 10, identifying conditions consistently associated with substantially elevated risk of cerebral palsy across epidemiological studies (1, 15, 19–21). This OR threshold was applied as a pragmatic decision to prioritize infants with significant biological vulnerability while limiting inappropriate over-referral. Consistent with contemporary diagnostic frameworks emphasizing proactive surveillance of infants with major neurological risk factors (1) and the BCCPAC's consensus recommendations (15), this approach supported a transparent and reproducible screening framework.

Therefore, eligible medical risk factors for referral to the EMSP included:

- Prematurity (< 32 weeks' gestation)
- Low birth weight (< 1,500 g)
- Cystic periventricular leukomalacia
- Grade III–IV intraventricular hemorrhage
- Moderate to severe neonatal encephalopathy (including hypoxic–ischaemic and infectious encephalopathy)
- Neonatal or postnatal meningitis
- Congenital central nervous system malformations
- Severe traumatic brain injury (< 4 months corrected age)
- Genetic diagnoses associated with CP
- Placental abruption
- Apgar score < 7 at 5 min
- History of stroke.

The inclusion of these medical risk factors enabled the EMSP to move beyond narrowly defined sentinel conditions toward a broader, biologically grounded model of early motor surveillance, strengthening the program's capacity to identify infants at elevated neurological risk during critical early windows for diagnosis and intervention.

The EMSP was launched in June 2022 at Sunny Hill Health Center, BC Children's Hospital, and delivered by a multidisciplinary team of GMA-trained occupational and physical therapists and developmental pediatricians. Initially, GMAs were conducted in person for infants meeting three sentinel high-risk criteria: neonatal stroke, severe hypoxic–ischaemic encephalopathy, or neonatal meningitis. While this model supported close interdisciplinary collaboration and high-quality assessment, it revealed substantial geographic and logistical barriers for families outside the Lower Mainland. Within 3 months of program launch, the EMSP transitioned to a fully virtual model to improve accessibility for families across BC, particularly those in rural, remote, and underserved regions. Concurrently, eligibility criteria were broadened to include all infants with identified medical risk factors, reflecting the BCCPAC's recommendation to move beyond restrictive neonatal follow-up criteria toward a proactive and inclusive approach to early identification.

Partnerships with Perinatal Services BC (PSBC) strengthened referral pathways to the EMSP with neonatal intensive care units across the province, and subsequent expansion enabled allied health professionals and birthing providers to refer directly. Provincial data indicate that approximately 700–800 infants meeting EMSP high-risk criteria are born annually in BC, yet historically only an estimated 150–200 received GMA through neonatal follow-up programs, underscoring a substantial gap in access to standardized early motor screening. Referral volumes to the EMSP therefore reflect both underlying epidemiological risk and health system factors such as clinician awareness, referral practices, and service accessibility, rather than population-level incidence.

When a submitted GMA video indicated absent or abnormal fidgety movements, suggesting a higher likelihood of CP, a therapist scheduled a virtual appointment with the child's caregivers to discuss the results and outline next steps. The discussion was guided by a modified SPIKES framework (Setting, Perception, Invitation, Knowledge, Explore emotions, and Strategy/Summary) (22), a structured method for delivering sensitive information with clarity and compassion. During the appointment, the therapist explained the assessment findings, discussed the implications of positive GMA results, and outlined the recommended care pathway, including referral to the BCCH Cerebral Palsy Early Diagnosis Clinic (CPEDC) when indicated. The therapist also addressed caregiver questions, provided anticipatory guidance, and offered practical strategies for home-based early intervention while families awaited formal diagnostic assessment. Following each appointment, families received a written summary that reinforced key messages and next steps, supporting continuity of care. The Cerebral Palsy Early Detection Clinic (CPEDC) provided an in-person diagnostic assessment following the Early Motor Screening Program for infants identified as having absent or abnormal General Movements Assessment results. Infants referred through the EMSP were scheduled for assessment when they reached approximately 5 months corrected age, typically 2 months following completion of the GMA screening.

CPEDC assessments are delivered through dedicated in-person clinics and are conducted by an interdisciplinary team consisting of a developmental pediatrician, an occupational therapist and a physiotherapist. Appointments are approximately 2 h in duration and include a comprehensive review of medical history, a structured caregiver interview focused on developmental history, and administration of the Hammersmith Infant Neurological Examination. Together, the EMSP and CPEDC form an integrated screening and assessment pathway, linking early remote identification with standardized, in-person neurological evaluation.

## Screening outcomes and pathway performance

Since implementation, the EMSP has expanded access to standardized early motor screening for infants across all health authorities in British Columbia, including urban, rural, and remote communities. Referrals were received from neonatal intensive care units (NICUs) of all levels, community pediatric practices, and specialized early intervention programs, demonstrating broad system uptake. Families residing outside major metropolitan areas accounted for a substantial proportion of referrals, highlighting the program's capacity to support equitable provincial access to early detection services. Results are presented descriptively to characterize implementation outcomes and clinical pathways rather than to evaluate diagnostic accuracy. The EMSP was not designed as a diagnostic test but as a pathway for early identification and triage within a high-risk population.

**Figure 1:**
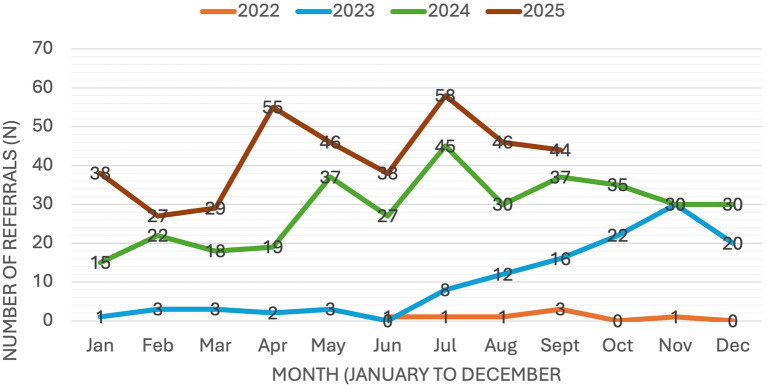
Monthly referral trends to the EMSP (June 2022–September 2025).

Referral volumes increased substantially over the 4-year period, with the most pronounced growth occurring between 2023 and 2025 (Figure 1). During the initial implementation phase in 2022, referral volumes were intentionally limited to support program initiation. Referral activity remained low in early 2023 and increased following expansion of clinical capacity in mid-2023. The addition of a second therapist supported a steady rise in referrals, reflecting both expanded service capacity and increasing provincial awareness of the program.

In 2024, referrals became more consistent, ranging from 15 to 45 per month (mean 26). Growth continued into 2025, with monthly referrals ranging from 27 to 58 and stabilizing between 55 and 58 from April to August. The 2025 mean of approximately 42 referrals per month represents a 62% increase compared with 2024 and a fourfold increase compared with 2023 (Table 1). Referrals were received from all regions of BC, indicating broad provincial participation.

**Table 1:** Annual referral trends to the early motor screening program.

**Year**	**Monthly range**	**Mean monthly referral**	**Total referrals per year**
2022	0–3	1	12
2023	0–30	10	205
2024	15–45	26	358
2025	27–58	42	389^*^ (on track to 512)

^*^2025 data through to September.

**Figure 2:**
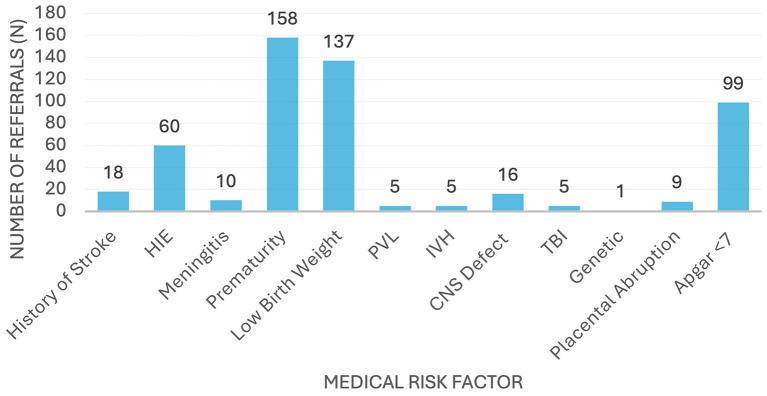
Distribution of medical risks factors referred to EMSP from 2022–24 (*N* = 523).

Between 2022 and 2024, 523 infants were referred to the EMSP based on medical risk factors (Figure 2). The most common risk factors were prematurity (*n* = 158; 30.2%), low birth weight (*n* = 137; 26.2%), and Apgar score < 7 at 5 min (*n* = 99; 18.9%), together accounting for more than 75% of referrals. Other notable risk factors included hypoxic–ischaemic encephalopathy (HIE; *n* = 60; 11.5%) and congenital central nervous system abnormalities (*n* = 16; 3.1%). Less frequent risk factors included stroke (*n* = 18), meningitis (*n* = 10), placental abruption (*n* = 9), periventricular leukomalacia (*n* = 5), intraventricular hemorrhage (*n* = 5), traumatic brain injury (*n* = 5), and genetic conditions (*n* = 1). A total of 180 infants (34%) presented with two or more medical risk factors.

Between June 2022 and September 2025, the EMSP received 883 referrals, of which 622 infants (70%) completed GMA assessments. The remaining 261 infants (30%) had not completed GMA at the time of reporting. The largest proportion of non-completed assessments comprised infants referred early who were awaiting age-appropriate timing for assessment within the fidgety period. Other reasons for non-completion included difficulty contacting families, redirection to neonatal follow-up services, infant medical instability or hospitalization, and late referral with insufficient time for assessment.

Among the 622 infants with completed assessments, 114 (18%) demonstrated abnormal GMA findings and were referred to the Cerebral Palsy Early Detection Clinic (CPEDC), while 508 (82%) had normal GMA findings and were discharged and an additional eight infants were referred directly to CPEDC without video submission due to late referral beyond the fidgety window.

Of the 114 infants referred to CPEDC following abnormal GMA findings, 25 (22%) received a diagnosis of cerebral palsy. 22 infants (28%) required ongoing developmental monitoring, and 20 infants (18%) were classified as having a high probability of CP based on early clinical features. 17 infants (15%) were determined not to have CP, while 13 infants (11%) were awaiting diagnostic appointments and nine infants (8%) were discharged or lost to follow-up. These findings describe the clinical yield of abnormal GMA findings within a real-world early detection pathway. No formal diagnostic accuracy analyses (e.g., sensitivity, specificity, or predictive values) were undertaken; therefore, observed proportions should be interpreted descriptively rather than as measures of test performance.

In parallel with program-level evaluation, provincial data indicate a temporal reduction in the average age of CP diagnosis among high-risk infants engaged in early detection pathways in British Columbia, decreasing from approximately 25 months to 7 months between 2022 and 2025 (8). This change reflects multiple coordinated early detection initiatives rather than the EMSP alone and should be interpreted as a system-level trend rather than a causal effect of the program.

The EMSP delivery model was designed to maximize clinical efficiency while maintaining quality of care. Caregiver-recorded videos reduced clinician assessment time by approximately 60 min per case, corresponding to an estimated 630 clinician hours saved during the study period. Operational performance was monitored through referral processing times, virtual appointment wait-times, caregiver experience measures, stakeholder feedback, and clinical pathway outcomes. Importantly, the findings of this study primarily reflect implementation and pathway performance rather than clinical efficacy, highlighting the feasibility and system-level implications of integrating early motor screening into provincial care pathways. Turnaround time from video submission to caregiver feedback was monitored as an implementation indicator but is not reported in detail in this study.

## Discussion

Guided by a family-centered approach and a Plan–Do–Study–Act (PDSA) quality improvement framework, the EMSP evolved through iterative refinements informed by caregiver and clinician feedback. Program materials were revised to improve clarity around video recording and upload procedures, and the initial telephone introduction was enhanced to better prepare families for the assessment process. These adaptations highlight the importance of responsive program design in virtual screening models and align with implementation science principles emphasizing iterative adaptation, stakeholder engagement, and system-level integration of evidence-based practices within real-world clinical contexts.

During program development, the term “cerebral palsy” was initially omitted from caregiver-facing materials to minimize distress among families, given that approximately 70%−80% of infants identified through medical risk factors do not ultimately receive a CP diagnosis. Feedback from caregivers revealed heterogeneous preferences regarding the amount of information provided at referral. In response, the EMSP adopted a flexible communication approach, allowing information to be tailored to individual family needs. This finding underscores the need for nuanced communication strategies in early detection pathways, particularly when screening occurs in contexts of clinical uncertainty.

Equity was a central consideration in the EMSP's design and implementation. The program sought to ensure that infants at elevated neurological risk could access timely assessment regardless of geographic location, socioeconomic circumstances, or local service availability. For families facing barriers to travel for in-person diagnostic assessment, EMSP clinicians facilitated early linkage to local therapy services while awaiting CPEDC appointments. This approach mitigated delays in intervention and provided families with interim support during periods of diagnostic uncertainty. The time-sensitive nature of the GMA (9–20 weeks corrected age) (10) posed specific implementation challenges. Missed or delayed assessments were primarily associated with late referrals, social or logistical barriers to video recording, and infant medical instability. Program adaptations included fast-track referral pathways for older infants, collaboration with local clinicians to support video capture, and direct referral to CPEDC in cases of significant clinical concern despite incomplete GMA data. Additional support was provided to families experiencing technological barriers, reinforcing the program's commitment to equitable access. At the same time, reliance on digital platforms may introduce new forms of inequity for families with limited technological access or digital literacy, underscoring the need for targeted supports within virtual screening models.

Although not a primary outcome, the virtual components of the EMSP may confer additional system-level benefits. To contextualize potential system-level co-benefits of virtual delivery, illustrative estimates of avoided travel were generated using program encounter data and published Canadian estimates (23–25). Between July 2022 and December 2024, the EMSP avoided an estimated 101,856 km of travel and at least 883 h of travel time, based on caregiver postal code data. Approximately 18% of families would otherwise have required more than 4 h of round-trip travel. These findings are not intended as formal environmental evaluations but rather as hypothesis-generating indicators of potential access and sustainability related co-benefits associated with virtual screening models.

Despite efforts to expand early identification pathways, clinician hesitancy persisted in some regions. This likely reflects a combination of unfamiliarity with the EMSP, competing clinical demands, and variable experience with early CP diagnosis. Capacity constraints within NICU workflows further contributed to inconsistent referral patterns. To address these barriers, the EMSP team implemented a multifaceted engagement strategy, including structured educational sessions, direct support for identifying eligible infants, strengthened communication with regional teams, and collaboration with Perinatal Services BC to standardize referral processes across the province. Partnerships with community pediatricians supported identification of older infants and toddlers with emerging motor concerns, complementing neonatal-focused pathways (26).

In the pragmatic context of real-world implementation, incomplete assessments reflected a combination of clinical, social, and system-level factors rather than selective exclusion based on clinical presentation. Formal comparative analyses between infants with completed and incomplete GMA assessments was outside of the scope of this paper. Consequently, reported proportions of abnormal GMA findings and downstream diagnostic outcomes apply only to infants with completed assessments and should be interpreted descriptively. The potential for systematic differences between screened and unscreened infants represents an important limitation and may influence estimates of screening yield.

Emerging digital health technologies may further strengthen early detection pathways. Automated identification of eligible infants through electronic health records, provincial dashboards monitoring screening outcomes, and machine-learning approaches to support GMA interpretation represent potential avenues for improving consistency and scalability (27). However, implementation in British Columbia is challenged by fragmented health information systems across multiple regional authorities. Greater interoperability and standardization could enhance referral efficiency and coordination across the province.

While developed within the context of British Columbia's publicly funded health system, the EMSP model may be particularly relevant to jurisdictions seeking to expand early CP detection beyond neonatal follow-up populations through virtual, risk-stratified, and interdisciplinary pathways. Within the international landscape of CP early detection initiatives, the provincial EMSP–CPEDC model operationalises pathway-based principles at a health-system level by integrating risk-based eligibility criteria, virtual screening, and coordinated interdisciplinary assessment within a publicly funded care structure, thereby extending existing approaches beyond predominantly clinic-based or research-driven models and addressing persistent gaps in access and equity.

This community-based implementation case study has several limitations. The evaluation was descriptive and not designed to test hypotheses, establish causality, or evaluate diagnostic accuracy. Observed associations between early screening, referral patterns, and diagnostic outcomes should therefore be interpreted as contextual rather than causal. Referral volumes reflect both underlying population risk and health system factors, including clinician awareness and service availability, and do not represent population-level incidence of CP risk. Incomplete GMA assessments limited comparative analyses and may have influenced estimates of screening yield. Abnormal GMA findings were reported descriptively, without formal diagnostic performance metrics. Observed reductions in the average age of CP diagnosis may reflect concurrent system-level changes rather than the EMSP alone. Finally, estimates related to clinician time savings, caregiver burden, and environmental impact were illustrative rather than based on formal economic or environmental modeling.

## Conclusion

The EMSP demonstrates that a province-wide virtual, selective screening model for infants at elevated neurological risk is feasible and scalable within a publicly funded health system. Through an integrated telehealth approach, standardized caregiver guidance, and structured communication processes, the program expanded access to high-quality GMA assessments across a geographically diverse population. The EMSP may mitigate access-related caregiver burden by reducing travel requirements and improving timeliness of assessment, while supporting improved equity for families with limited access to specialized services and strengthened provincial alignment around early detection pathways.

Findings from the first 3 years highlight the importance of flexible, family-centered communication, streamlined referral processes, and sustained collaboration with NICUs, community pediatricians, and provincial agencies. Ongoing implementation challenges underscore the need for continued system-level coordination and targeted provider education. Emerging digital tools, including automated referral prompts and machine-learning approaches to support GMA interpretation, represent promising opportunities to enhance consistency, efficiency, and scalability in future iterations of early detection pathways.

Provincial data further indicate a temporal reduction in the average age of CP diagnosis among high-risk infants engaged in early detection pathways in British Columbia, from approximately 24 months to 7 months during the study period. This change reflects multiple concurrent system-level initiatives rather than the EMSP alone but is consistent with the potential value of coordinated early detection pathways in supporting more timely identification within real-world clinical contexts.

Overall, the EMSP provides a practical and adaptable model for jurisdictions seeking to strengthen early CP detection beyond traditional neonatal follow-up populations. Continued quality improvement, system integration, and evaluation will be essential to sustaining long-term impact and ensuring equitable access to early motor screening and follow-up care for infants and families across British Columbia and beyond.

## Data Availability

The original contributions presented in the study are included in the article/Supplementary material, further inquiries can be directed to the corresponding authors.
