# Supplementary figures and images for: Implementation of an innovative virtual selective screening program for early detection of cerebral palsy in British Columbia
Source: Front Public Health. 2026 Mar 2;14:1754120. doi: 10.3389/fpubh.2026.1754120 (PMC12989511; doi:10.3389/fpubh.2026.1754120)

Appendix:


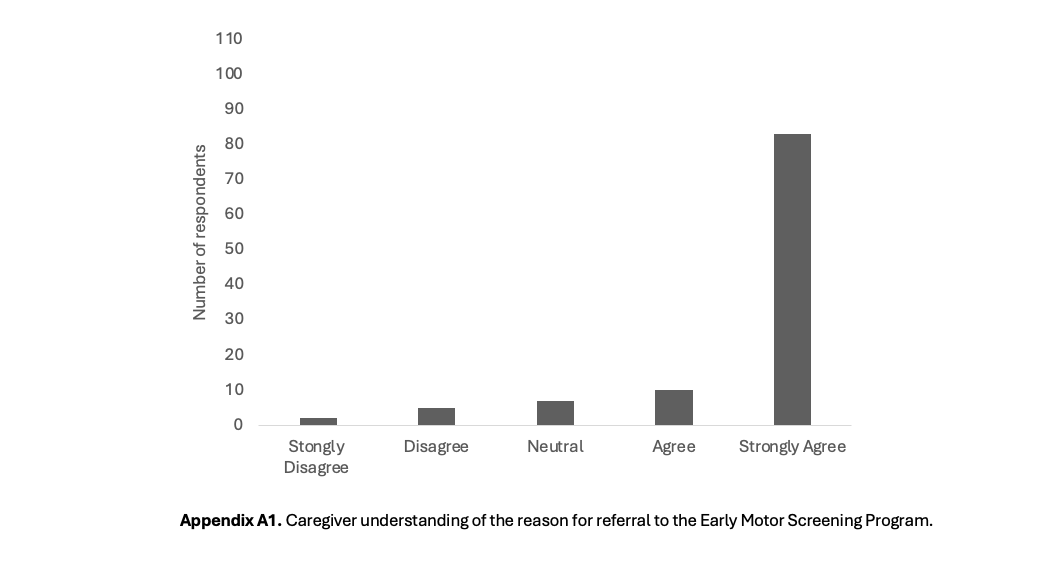


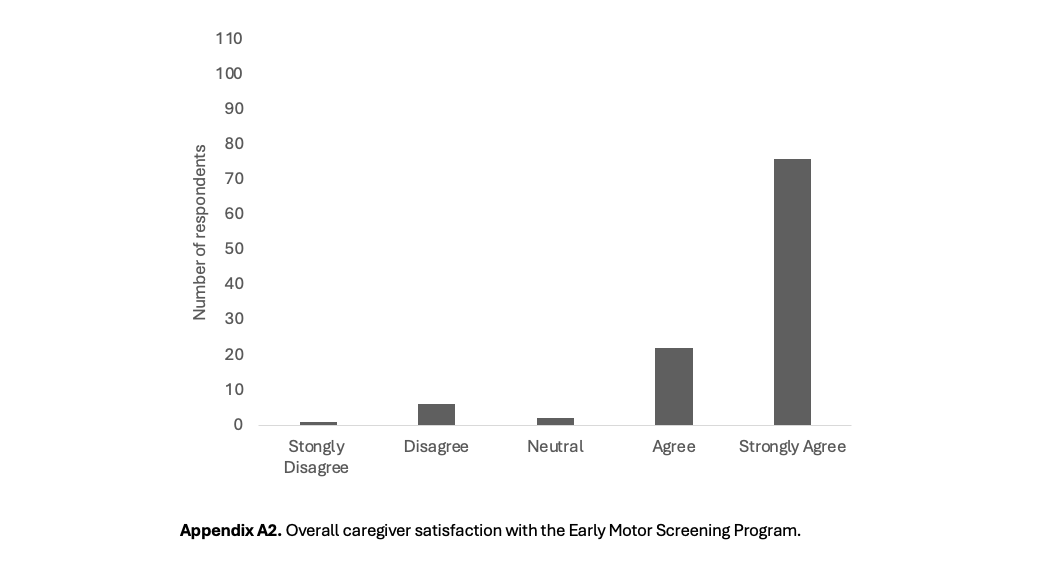


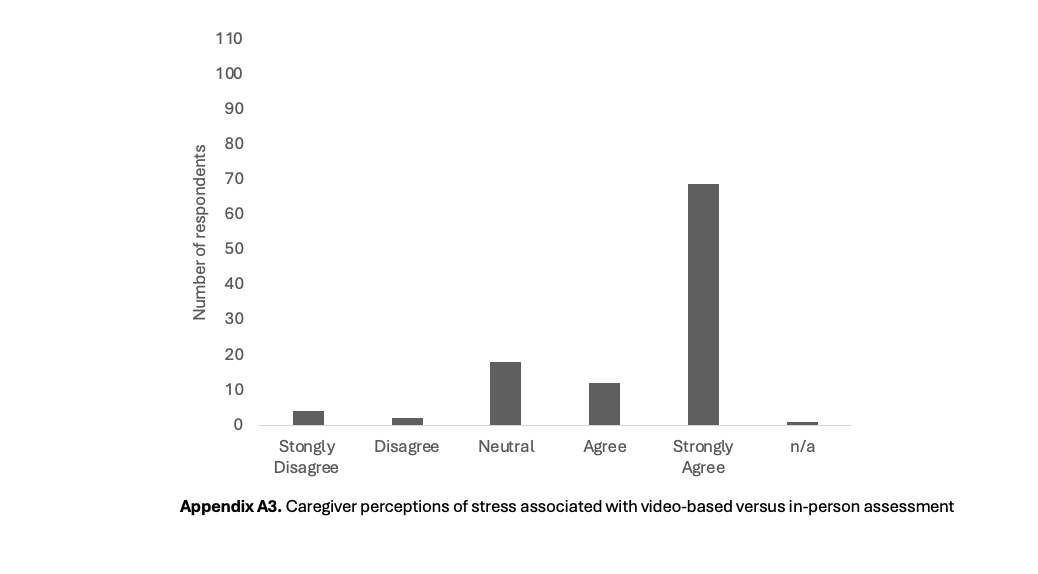


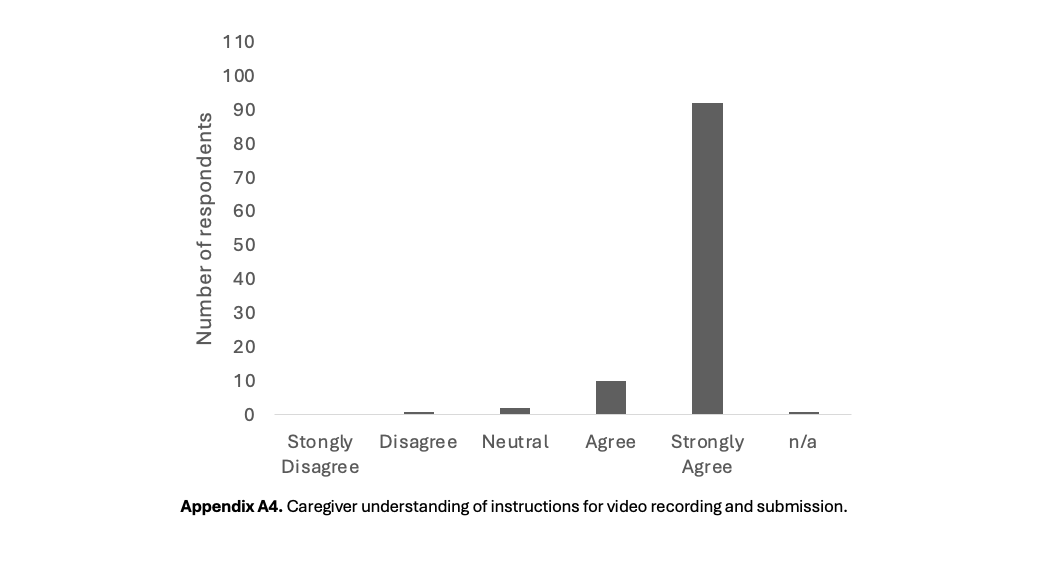


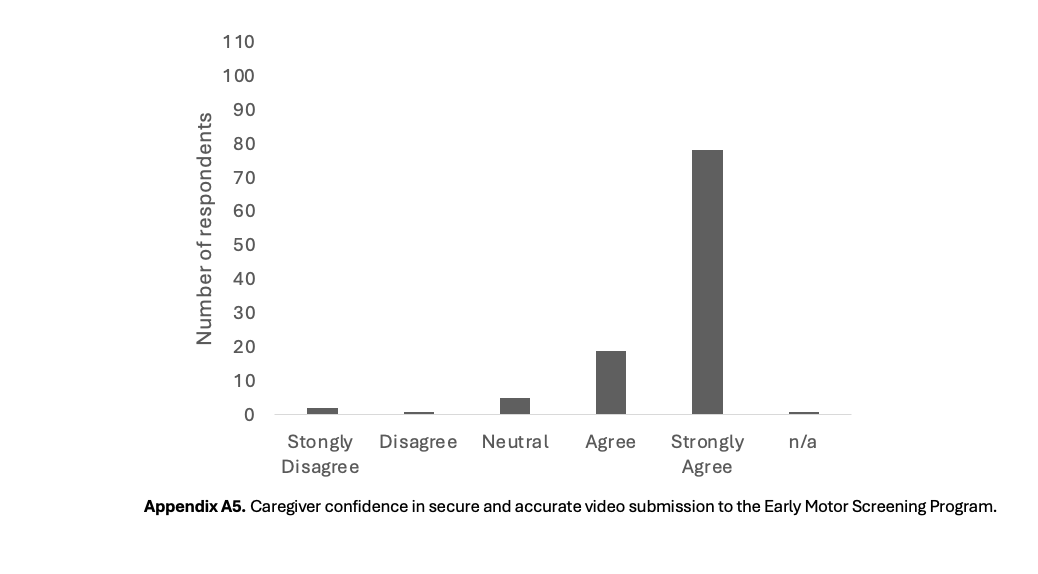

Supplement: Supplementary file 1 [file Data_Sheet_1.docx]
